# Protein Transduction Domain Mimic (PTDM) Self-Assembly?

**DOI:** 10.3390/polym10091039

**Published:** 2018-09-19

**Authors:** Nicholas D. Posey, Gregory N. Tew

**Affiliations:** 1 Department of Polymer Science and Engineering, University of Massachusetts Amherst, Amherst, MA 01003, USA; nposey@mail.pse.umass.edu; 2 Department of Polymer Science and Engineering, Department of Veterinary and Animal Sciences, Molecular and Cellular Biology Program, University of Massachusetts Amherst, Amherst, MA 01003, USA

**Keywords:** block copolymers, interfacial tension (IFT), self-assembly, TEM, protein transduction domain mimics, antibodies, dynamic light scattering (DLS)

## Abstract

Intracellular protein delivery is an invaluable tool for biomedical research, as it enables fundamental studies of cellular processes and creates opportunities for novel therapeutic development. Protein delivery reagents such as cell penetration peptides (CPPs) and protein transduction domains (PTDs) are frequently used to facilitate protein delivery. Herein, synthetic polymer mimics of PTDs, called PTDMs, were studied for their ability to self-assemble in aqueous media as it was not known whether self-assembly plays a role in the protein binding and delivery process. The results obtained from interfacial tensiometry (IFT), transmission electron microscopy (TEM), transmittance assays (%T), and dynamic light scattering (DLS) indicated that PTDMs do not readily aggregate or self-assemble at application-relevant time scales and concentrations. However, additional DLS experiments were used to confirm that the presence of protein is required to induce the formation of PTDM-protein complexes and that PTDMs likely bind as single chains.

## 1. Introduction

The initial discovery of a cell-membrane penetrating protein called HIV-1 tat in 1988 has given rise to the modern field of cell-penetrating peptides (CPPs) and protein transduction domains (PTDs), which now include cell-membrane-penetrating transporters such as transportan, Pep-1, penetratin, CADY, and MPG among others [1,2,3,4,5,6,7,8,9]. CPPs and PTDs are often utilized for biomedical applications given that their cell penetrating function facilitates the delivery of exogenous biologics, such as nucleic acids and proteins, into cells both in vitro and in vivo [10]. The field of polymer chemistry has capitalized on key CPP structure-activity studies to produce effective polymer mimics, known as CPPMs and PTDMs. The guanidinium groups in CPPs and PTDs are the most frequently mimicked feature, appearing often in their synthetic polymer counterparts [4,11,12,13,14,15,16].

Though other groups have designed CPPMs and PTDMs through a variety of polymerization techniques, our group has focused specifically on PTDM design by leveraging a ring-opening metathesis polymerization (ROMP) platform [4,13,16,17,18,19]. Dual functional, oxanorbornene ROMP-monomers and the well-controlled nature of the polymerization have allowed for the synthesis of highly tuned and optimized amphiphilic block copolymer PTDMs, where parameters such as guanidinium density, hydrophobic side chain density, overall hydrophobicity, block length, block ratios, and block segregation can be manipulated [20,21,22,23,24,25,26,27,28]. Since the first PTDM developed by our group in 2008, we have thoroughly explored the PTDM synthetic parameter space and used those well-defined polymers to deliver bioactive siRNA and functional protein cargo, including antibodies, into difficult-to-transfect T cells, among other cell types [20,21,23,29,30,31]. Additionally, our amphiphilic block copolymer PTDMs have consistently found greater success as delivery reagents than their cationic homopolymer counterparts [24,29].

Amphiphilic block copolymers are especially known for their ability to self-assemble in solution as well as in the bulk [32]. Often water-soluble, amphiphilic copolymers are characterized for their surfactant-like qualities, using interfacial tensiometry (IFT), and for their propensity to self-assemble, using dynamic light scattering (DLS) or transmission electron microscopy (TEM) [33,34,35,36]. Given that our PTDMs have distinct hydrophilic and hydrophobic blocks and that our PTDMs are used in applications involving aqueous media, a reasonable assumption would be that these polymers undergo some self-assembly or aggregation. For that reason, our low dispersity (Đ ~ 1.1) block copolymer PTDMs (~6000 g/mol for PTDM1) [21], which have molecular weights higher than that of small molecule surfactants, like sodium dodecyl sulfate (SDS) (288.38 g/mol), but lower than that of traditional amphiphilic block copolymers, have been studied for their ability to self-assemble by DLS previously, but at elevated concentrations (2.5 mg/mL or 5 mg/mL), far above application-relevant conditions [20]. Questions still remain about PTDM self-assembly and whether this has any bearing on their ability to bind and deliver protein cargo. For example, in a previous report, PTDM1 (structure shown below) was present at a concentration of ~36 µg/mL to form complexes with enhanced green fluorescent protein (EGFP) in solution before dilution down to ~7 µg/mL, with respect to PTDM1, for the protein delivery step [21]. Due to these low concentrations, which complicate characterization techniques such as light scattering, the formation of PTDM-protein complexes has been characterized using transmittance (%T) assays by our group, as well as by using fluorescence-based binding assays that enable the calculation of dissociation constants [21,28,31,37].

Herein, the ability of our PTDMs to spontaneously self-assemble is revisited at application-relevant concentrations. A combination of characterization techniques, including pendant drop IFT, DLS, %T assays, and TEM, were employed to investigate the propensity of our PTDMs to self-assemble. Overall, our findings suggest that PTDMs are not inclined to form robust and stable self-assembled structures at low, application-relevant concentrations, though some data seem to indicate that PTDMs are capable of forming aggregates in phosphate buffered saline (PBS) over long time periods.

## 2. Materials and Methods

### 2.1. Materials

Dimethyl sulfoxide (DMSO) was purchased from Fisher Scientific. PBS buffer, 10×, pH 7.4, was purchased from Thermo Fisher Scientific, Inc. (Waltham, MA, USA) which was diluted to 1× with Milli-Q^®^ water and adjusted to pH 7.2 with TITRISTAR^®^ 0.1 N hydrochloric acid from EMD Chemical, Inc. (Gibbstown, NJ, USA) and an Accumet^®^ Basic AB15 pH meter from Fisher Scientific prior to use. The Milli-Q^®^ water used for all TEM, IFT, Plate Reader, and DLS experiments was obtained by further purifying house reverse osmosis (RO) water using a Millipore Milli-Q^®^ UF plus system (Burlington, MA, USA). After storage, the pH of this Milli-Q water was measured to be between 4 and 5 by pH indicator strips (pH range of pH 0–14 from EMD Chemical Inc.). All materials required for the synthesis of PTDM1 have been previously reported [20,21,29]. For interfacial tensiometry (IFT) measurements, Optima^®^ HPLC grade Toluene was obtained from Fisher Scientific. In addition, an optical glass cuvette with a total cell volume of 3.5 mL was obtained from Science Outlet (Hong Kong Special Adminstrative Region, Hong Kong, China) and a flat-ended metal needle (22 gauge, 0.718 mm nominal outer diameter with a Kel-F hub) was obtained from the Hamilton Company (Reno, NV, USA). Plastic, disposable, NORM-JECT^®^ 1 mL syringes came from Henke-Sass, Wolf GmbH (Tuttlingen, Germany). For transmission electron microscopy (TEM) experiments, carbon films on copper grids (400 mesh, ultra-thin) were purchased from Electron Microscopy Sciences (Hatfield, PA, USA). In addition, KIMTECH^®^ Kimwipes Science Brand came from Kimberly Clark Professional (Roswell, GA, USA) and anti-mouse IgG (whole molecule)-FITC produced in goat, affinity isolated antibody (IgG-FITC) was purchased from Sigma-Aldrich (St. Louis, MO, USA) as an aqueous buffered solution. For plate reader experiments, 96 well UltraCruz^®^ UV plates with clear, flat bottoms were purchased from Santa Cruz Biotechnology (Dallas, TX, USA). For dynamic light scattering (DLS) experiments, disposable poly(styrene) cuvettes, with a cell volume capacity of 4.5 mL, were purchased from Fisher Scientific. In addition, green PES syringe filters (size 13 mm, pore size 0.45 µm) came from the Restek Corporation (Bellefonte, PA, USA). PARAFILM^®^ “M” laboratory film came from Pechiney Plastic Packaging (Bemis NA, Neenah, WI, USA).

### 2.2. Instrumentation and Software

IFT experiments were conducted using a Contact Angle System OCA 15plus made by Dataphysics Instruments GmbH (Filderstadt, Germany). Interfacial tension as a function of time was calculated by fitting the suspended droplet shape to the Young-Laplace equation using a SCA22 “surface and interfacial tension” software module. TEM images were collected using a FEI Technai T12 TEM equipped with a TVIPS TemCam-F216 camera and using EM-Menu4 software (version 4.0.9.83, Gauting, Germany). DLS experiments were conducted using a Malvern Instruments Zetasizer Nano ZSP and data analysis was done with the corresponding zetasizer software (version 7.11, Westborough, MA, USA). Light scattering was measured at a back-scattering angle of 173° (the NIBS default instrument setting). The standard operating procedure (SOP) used for all DLS experiments included setting poly(norbornene) as the “material” where the refractive index (RI) was estimated to be 1.5. The “dispersant” selected in the SOP was either water (instrument preset, RI = 1.330) or a “complex solvent”, PBS, (RI = 1.332, viscosity = 0.9071 cP), which was custom built using estimates provided by the software solvent builder module, where the primary component selected was water and the minor components consisted of sodium chloride (0.1552 mol/L), sodium phosphate dibasic (0.0030 mol/L), and sodium phosphate (0.0011 mol/L) as a substitute for potassium phosphate monobasic, which was the actual component in the PBS. The disposable cuvette option DTS0012 was also selected in the SOP. All plate reader experiments were conducted using a BioTek SynergyMx plate reader with Gen5™ software (version 1.10.8, Winooski, VT, USA). For %T assays, the pathlength correction feature was selected in the software. For the collection of UV-visible spectra, the software was programmed to scan every wavelength in increments of 1 nm between 230 nm to 750 nm (read speed normal setting).

### 2.3. PTDM Selection and Stock Solution Preparation

PTDM1 was chosen for this study as the model PTDM, since its synthesis and characterization had been previously reported [20,21,29]. The theoretical molecular weight of PTDM1, based on its idealized, stoichiometric structure is 5899.25 g/mol which was used for calculations to create a 1 mM stock solution in DMSO [21,29]. The PTDM1 stock dissolved in DMSO was stored at −20 °C, causing it to freeze. Prior to use, the stock solution was allowed to thaw and reach room temperature after which time it was vortexed for ~30 s. For all experiments, the 1 mM PTDM1 stock solution in pure DMSO was always diluted with either Milli-Q^®^ water or 1× PBS to obtain the lower concentrations required for a given experiment, a process which always led to residual amounts of DMSO in the final PTDM1 solution depending on the extent of dilution. For example, all 10 µM solutions of PTDM1 had 1% *v*/*v* residual DMSO whereas 100 µM solutions of PTDM1 had 10% *v*/*v* residual DMSO.

### 2.4. Pendant Drop Interfacial Tensiometry (IFT)

PTDM1 solutions at 1, 10, and 100 µM were prepared via serial dilution of a 1 mM PTDM1 stock solution in pure DMSO. DMSO was added to the 1 and 10 µM PTDM1 solutions such that all PTDM1 solutions tested contained 10% DMSO *v*/*v*. Control solutions of pure Milli-Q^®^ water and Milli-Q^®^ water with 10% *v*/*v* DMSO were also prepared. A flat-ended needle and a 1 mL disposable, plastic syringe were used to draw up the aqueous phase. The 1 mL syringe was then loaded into the syringe pump portion of the instrument where it was secured. Manual use of the syringe pump was required to expel air from the tip of the needle prior to submersion of the needle into a Toluene ambient phase (~3 mL), which was contained within an optically clear, glass cuvette. After proper alignment and focusing of the instrument camera on the needle, the automated syringe pump control was used to dispense a droplet such that a large, tear-shaped droplet hung from the needle. The following reference was consulted with respect to obtaining a proper drop shape and size [38]. Usually, the first droplet was discarded, and the formation of a second, suitable droplet was followed by data collection (droplet imaging with the instrument/camera set to take 1 measurement per second). The software kept time such that *t* = 0 on the raw data plots refers to when the data collection started, not the time at which the droplet was first formed, but only equilibrium IFT values away from *t* = 0 were reported and used for calculations. The temperature of the room (usually between 21 and 22 °C) and the manufacturer-reported outer diameter of the needle was input into the software interface, along with the identity of both phases using presets in the instrument software, which then set the density of the phases based on their identity. The manufacturer-reported outer needle diameter of 0.718 mm, which was also verified manually with calipers to be between 0.71–0.72 mm, was used as the input parameter.

Each trial run was stopped once equilibrium values for IFT had been achieved as shown in Figures S1–S5. A single, average IFT value for a given sample was generated by averaging all data points obtained from all trials from the stable, equilibrium portion of the curve as discussed in Figures S1–S5. The change in IFT, denoted as ΔIFT, was calculated as IFT_Sample_ − IFT_Control_ in an attempt to show by how much each surfactant sample, including examples from literature, changed the IFT relative to their respective controls and each other. All IFT values used in calculations for all surfactants from literature, all PTDM1 solutions, and their respective controls are summarized in Table S1.

### 2.5. Transmission Electron Microscopy (TEM)

A 1× PBS solution with 1% *v*/*v* DMSO was prepared. In separate experiments, a 10 µM PTDM1 solution in Milli-Q^®^ water which contained 1% *v*/*v* residual DMSO was prepared from the aforementioned 1 mM stock described above. A 200 nM IgG-FITC antibody solution was also prepared by diluting a commercially obtained 1.1 mg/mL antibody (Ab) solution with Milli-Q^®^ water. The theoretical Ab molecular weight used for the calculation was 151,479.64 g/mol, which took into account the number of FITC fluorescent dyes on the Ab, which was 3.8 on average, according to the manufacturer.

To prepare a dried TEM grid for imaging, a clean grid was taken with TEM tweezers and held while a pipette was used to dispense a 3 µL sized droplet onto the grid. After 30 s, the excess liquid on the grid was removed by touching the liquid on the surface of the grid with the edge of a clean KIMTECH^®^ Kimwipe. The grid was stored on filter paper and covered by a plastic box and then allowed to dry overnight in ambient conditions. After one night of drying, dried grids were stored in a separate TEM grid box until they were imaged. All grids were imaged using a FEI Technai T12 TEM.

### 2.6. Dynamic Light Scattering (DLS)

All DLS data were collected using a Malvern Zetasizer Nano ZSP set to measure back-scattered light at an angle of 173°. PTDM1 solutions at 10 µM in either PBS or Milli-Q^®^ water were prepared by diluting the aforementioned PTDM1 1 mM DMSO stock solution. The total volume of all solutions used for DLS experiments was set at 1 mL to satisfy the minimum solution level required by the instrument. All 1 mL solutions were filtered using 2 mL syringes to pass the sample solutions through poly(ethersulfone) (PES) filters. The excess air in the 2 mL syringe was also passed through the filter so that liquid left in the filter could be pushed into cuvette and recovered. All solutions were filtered directly into disposable poly(styrene) cuvettes, which had been washed both on the inside and outside with filtered methanol several times and blown dry with filtered air. All cuvettes were stored in clean foil pouches overnight prior to experiments to allow for complete drying and to prevent dust accumulation. For the DLS experiments done after a ~24 h waiting period, the sample preparation was identical except that after filtering directly into cleaned cuvettes, the top of the cuvettes was wrapped in PARAFILM^®^ to prevent evaporation of the samples. The samples were carefully wrapped in foil pouches and stored upright for ~24 h prior to DLS experiments.

### 2.7. DLS Titration Experiment

For the DLS titration experiment, a solution of IgG-FITC was prepared fresh at 200 nM, with respect to protein, from the commercially obtained protein solution. The molarity of the antibody solution was calculated by using 150,000 g/mol, as the base molecular weight of IgG, and by taking into account the molecular weight added by the conjugation of the FITC dye molecules, which varies depending on the lot/batch of protein. The product information and lot number specification sheets were consulted to determine at what concentration the original antibody solution was provided and how many dyes on average there were per protein. Once a solution was prepared, it was filtered directly into a prepared PS disposable cuvette as described above.

One milliliter of antibody-only solution was prepared in PBS, buffered at pH ~7.2, and DLS measurements were made using the aforementioned software parameters, except the generic “protein” “material” instrument preset was selected this time. This included the parameters RI (refractive index) = 1.45 and absorbance = 0.001. Three independent DLS experiments were done with three antibody-only solutions first to determine the size distribution by intensity and number as a baseline for the titration. As for the titration experiments, a fresh 200 nM antibody-only solution was prepared immediately before each titration. A baseline reading of the antibody-only solution was taken first to confirm that it matched the antibody positive control data. After the initial reading, aliquots of the PTDM1-only solution (100%) DMSO were added incrementally to the antibody-only solution and pipetted up and down and stirred gently. After mixing, the same original cuvette was checked for dust on the sides and placed back in the DLS instrument for measurements. This process was repeated until measurements had been taken for the following 10 µL additions and one final 20 µL resulting in 10 then 20 then 30, 40, 50, 60, 70, 80, and finally 100 total µL of added PTDM1-only solution. The experiment was stopped at 100 total µL PTDM1-only solution added because this amount of the solution resulted in a final concentration of PTDM1 of ~90.9 µM which was past the saturation point studied in the corresponding fluorescence quenching experiments. The titration experiment was repeated a second time. Additional information about this experiment can be found in the Supplementary Materials (Table S2).

### 2.8. Transmittance (%T) Assays and UV-Visible Spectra

%T time course curves and UV-visible spectra were obtained using a BioTek plate reader and UV transparent 96-well plates. PTDM1 solutions at a concentration of 10 µM (1% *v*/*v* residual DMSO) in both Milli-Q^®^ water and PBS with a total volume of 200 µL were made in the wells of the plate along with two control solutions, one being a 200 µL total volume solution of pure Milli-Q^®^ water and another being a 200 µL total volume solution of pure PBS. Prior to %T assays, UV-visible spectra were obtained at 25 °C for all solutions with optical density measurements taken every 1 nm between 230 and 750 nm. Transmittance (%T) was then determined by first measuring the absorbance (A) of four wavelengths of light (300, 400, 500, and 700 nm) that were passed through the solutions of the wells in the plate. The first measurement taken was considered *t* = 0, after which time the instrument was programmed to delay for two hours before taking another measurement, which was deemed *t* = 2 h. This cycle of %T measurements of all wells followed by a two-hour delay was repeated 12 times, such that the total delay time accrued was 24 h. The total experiment time took more than 24 h when the time of taking the measurements and reading the initial UV-visible spectra were taken into account. During this total time of over 24 h, the plate was stored immobile in the plate reader and was not disturbed. In terms of data processing, the absorbance (A) values of the control solutions were subtracted out of the sample solutions wells at all time points as background, and the remaining absorbance (A) due to the PTDM1 sample solution was converted to %T with the following equation: %T = 10^(2−A)^. All plate reader experiments were repeated to obtain a total of two independent trials.

## 3. Results and Discussion

**Figure 1:**
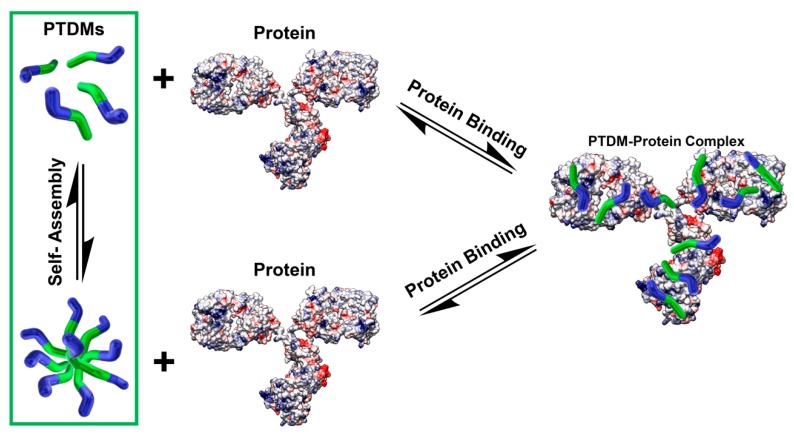
Two hypothesized pathways by which PTDMs could bind protein cargo: one involving single chains in solution and another involving self-assembly. The Coulombic surface potential model of immunoglobulin (Protein Data Bank (PBD) ID: 1IGT), shown without ligands, was generated using the UCSF Chimera package [40] and the corresponding .pdb file from PBD entry 1IGT [41]. Chimera software is developed by the Resource for Biocomputing, Visualization, and Informatics at the University of California, San Francisco (supported by NIGMS P41-GM103311).

The role of PTDM self-assembly in protein binding and delivery applications is unresolved. We hypothesize that there are two main pathways by which PTDMs could bind protein cargo *en route* to producing deliverable PTDM-protein complexes (Figure 1). One path involves PTDMs binding protein as single chains and another involves PTDMs self-assembling prior to interacting with the protein. Some surfactants are known to bind and denature protein, but we suspect that PTDMS do not induce gross protein denaturation given that they have delivered functional protein intracellularly in past reports [28,30,39]. In protein delivery applications, binding and PTDM-protein complex formation takes placed in PBS prior to delivery, whereas delivery takes place in complete media, which includes serum proteins. The focus of this communication is to understand self-assembly in the context of the protein binding step. PTDM1 was selected as the model PTDM here due to its previous success in protein binding and delivery applications. PTDM1 is also among the more hydrophobic variants demonstrating intracellular protein delivery [20,21,29,30,31].

### 3.1. Interfacial Tension (IFT)

**Figure 2:**
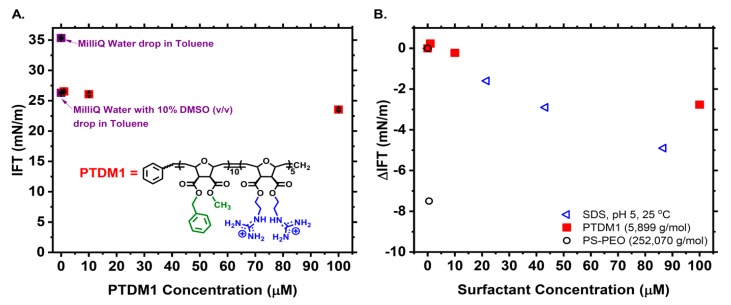
(**A**) Interfacial tension (IFT) measured for an aqueous phase droplet containing Milli-Q^®^ water, Milli-Q^®^ Water with 10% DMSO *v*/*v* (dark blue squares), or Milli-Q^®^ Water with 10% DMSO *v*/*v* and varying concentrations of PTDM1 (red squares) in a toluene ambient phase. Inset shows the chemical structure of PTDM1. (**B**) Change in interfacial tension (ΔIFT) between a control solution containing no PTDM1 and solutions containing varying concentrations of PTDM1 (red solid squares). Open data points were generated from literature values for a PS-PEO block copolymer or sodium dodecyl sulfate (SDS) at various concentrations as compared to their respective control solutions [36,42]. ΔIFT was calculated as IFT_Sample_ − IFT_Control_ for all surfactants. The theoretical molecular weight for PTDM1 is shown in the legend along with the calculated molecular weight of PS-PEO from literature.

To ascertain whether PTDMs have surfactant characteristics, which would promote self-assembly in aqueous media, pendant drop interfacial tension (IFT) studies were conducted in which an aqueous droplet of Milli-Q^®^ water, with 10% *v*/*v* DMSO, containing PTDM1 over a range of concentrations of 1, 10, and 100 µM, was suspended in a toluene ambient phase (Figure 2 and Figure S1–S5). The IFT was measured over time until it reached equilibrium for all samples (Figures S1–S5). The stable IFT values in the equilibrium range, from at least two independent trials, were then averaged to generate the one mean value plotted in Figure 2A. A control experiment that measured the IFT of a Milli-Q^®^ water droplet in a Toluene ambient phase, yielded an equilibrium IFT value of ~35 mN/m which was in agreement with literature values (Figure S1) [36,42]. A separate control in which the Milli-Q^®^ water droplet was made with 10% *v*/*v* DMSO showed that the presence of DMSO decreased the interfacial tension by ~9 mN/m, but to a new stable equilibrium value (Figure S2). This control was necessary because each PTDM1 solution tested also contained 10% *v*/*v* DMSO for consistency as the 100 µM PTDM1 solution contained 10% *v*/*v* residual DMSO as a result of its sample preparation.

Despite covering three orders of magnitude with respect to concentration, PTDM1 failed to act as a strong surfactant resulting in only a slight decrease in IFT even at its highest concentration, 100 µM (Figure S5). One hundred µM is more than ten times higher than the concentration of PTDM1 that was present for binding EGFP cargo and forming complexes in a previous report [21]. Over the application-relevant concentration range, 1 to 10 µM, the lack of surfactant character is evident. To fully illustrate the poor surfactant nature of PTDM1, the change in IFT, calculated as ΔIFT = IFT_sample_ − IFT_control_, was plotted in Figure 2B along with ΔIFT values calculated from literature for the PS-PEO block copolymer surfactant and sodium dodecyl sulfate (SDS) (Table S1) [36,42]. Comparing PTDM1 to the small molecule anionic surfactant, SDS, over a similar concentration range highlights that PTDM1 is a worse surfactant than SDS. In fact, SDS has a literature-reported critical micelle concentration (CMC) of ~8 mM [42]. This implies that PTDM1, being a worse surfactant, would have a higher CMC than SDS and not assemble in the delivery relevant concentration range of 1–10 µM. Likewise, the standard block copolymer surfactant PS-PEO decreases the IFT of a water-toluene interface much more than SDS or PTDM1 at lower concentrations of <1 µM. The PS-PEO polymer surfactant from literature has a significantly higher molecular weight than PTDM1, which could complicate direct comparisons. That very observation, however, emphasizes the point that in order to be appreciably surface active at low molar concentration, polymers seem to need to have higher molecular weights [33,36,43]. As for an example of a low molecular weight polymer surfactant, a PEO-PPO-PEO triblock, with *M*_n_ ~ 1460 g/mol and only 22 repeat units of PO and 4 repeat units of EO, was able to decrease the IFT of a water-toluene interface by 19.9 mN/m, as compared to its control, at a concentration of ~6.85 mM. Other small PPO-PEO diblocks from the same study were similarly effective at decreasing IFT in a similar mM concentration range (Table S1) [44]. These examples further illustrate the point that if a polymer surfactant has a low molecular weight, then it probably needs to be in higher molar concentrations to be interfacially active. As such, PTDM1’s approximate molecular weight (~6000 g/mol) is also low and could possibly account for PTDM1’s lack of interfacial activity at µM level concentrations. Overall, our evaluation of the IFT data suggests that PTDM1 exists as a single chain in water in the application-relevant concentration range of 1–10 µM and that it is not driven to self-assemble on account of its poor surfactant nature.

### 3.2. Transmission Electron Microscopy (TEM)

**Figure 3:**
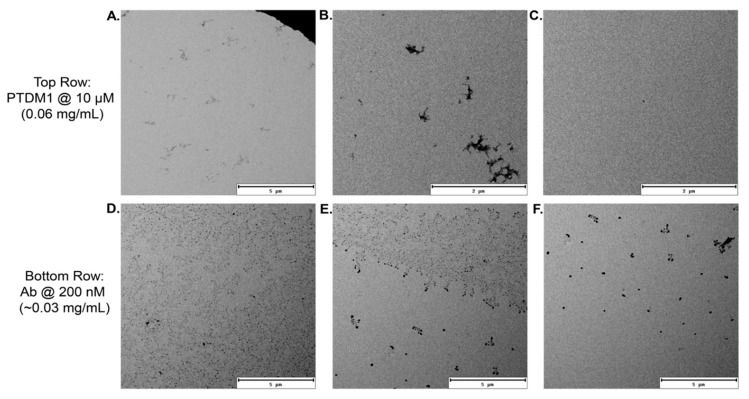
Transmission electron microscopy (TEM) micrographs of PTDM1 (**A**–**C**) and IgG-FITC whole antibody (Ab) (**D**–**F**) solvent cast from Milli-Q^®^ Water at 10 µM for PTDM1 and at 200 nM for the Ab on TEM grids which were dried in ambient conditions overnight. (**A**) Large area image of PTDM1 in focus showing large drying artifacts. (**B**) Large area image of PTDM1 at 2.50 µm under focus showing large, medium, and small drying artifacts. (**C**) Large area image of PTDM1 at 2.50 µm under focus showing a small drying artifact. (**D**) Large area image of Ab at 2.96 µm under focus showing an abundance of aggregated material. (**E**) Large area image of Ab at 2.94 µm under focus showing a sparser region of aggregated material. (**F**) Large area image of Ab in focus showing large drying aggregates.

TEM can be used to visualize self-assembled amphiphilic polymer structures even after drying samples on grids [33,45]. For this reason, PTDM1 was studied by TEM to assess whether it is capable of forming nanoscale or microscale aggregates. PTDM1 solutions at 10 µM in Milli-Q^®^ water were solvent cast onto TEM grids, had excess liquid removed, and were further dried in ambient conditions overnight. Figure 3A–C shows large area TEM images of PTDM1, in which only large and small drying artifacts can be seen. The process of drying the solutions likely caused the formation of the large, irregularly shaped aggregates, which was expected. The small, dark black dots seen in the images (example of one highlighted in Figure 3C) are standard small drying aggregates and not a self-assembled morphology according to the TEM literature [46]. To illustrate this point, 1× PBS with 1% *v*/*v* DMSO was cast onto TEM grids and allowed to dry overnight. The PBS-only sample also showed small black dot drying aggregates, demonstrating that this morphology could be reproduced by drying a salt solution (Figure S7).

It was difficult to find any evidence by TEM that PTDM1 had self-assembled into robust, stable structures capable of enduring the drying process. To alleviate the concern that TEM was incapable of visualizing material that had been solvent cast from low concentrations (0.06 mg/mL for PTDM1), another dried TEM sample was prepared with an IgG-FITC whole antibody (Ab) solution at a similarly low concentration of ~0.03 mg/mL. This sample resulted in the large area images shown in Figure 3D–F. The abundance of material seen in those large area images affirms that TEM is capable of detecting material residing on the grid at low concentrations. While the IgG-FITC solution did contain small amounts of residual salt from the commercially obtained protein solution, it is unlikely that those salts led to the abundance of detectable material seen in Figure 3D–F, as the PBS-only TEM sample contained significantly more salt and only resulted in the few small drying aggregates seen in Figure S7. The lack of self-assembled structures in the TEM images corroborates the data from the IFT experiments in that it supports the notion that PTDM1 is not prone to aggregate in aqueous environments at application-relevant concentrations.

### 3.3. Dynamic Light Scattering (DLS) and Transmittance Assays (%T)

**Figure 4:**
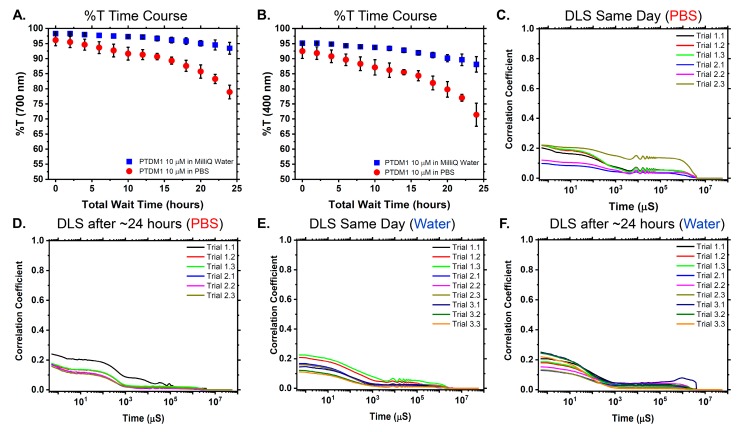
Characterization of PTDM1 in aqueous solutions using transmittance (%T) assays and dynamic light scattering (DLS). (**A**) %T of 700 nm light through solutions containing PTDM1 at 10 µM in both Milli-Q^®^ water (blue squares) and PBS (red circles). Results obtained are the average of two independent trials where the average %T is plotted and the error bars represent ± one standard deviation. (**B**) %T of 400 nm light through the same solutions containing PTDM1 at 10 µM in both Milli-Q^®^ water (blue squares) and PBS (red circles). Results obtained are the average of two independent trials where the average %T is plotted and the error bars represent ± one standard deviation. (**C**) DLS correlation coefficient plots for two independent trials of PTDM1 at 10 µM in PBS where each trial run was comprised of three measurements. Each sample was prepared immediately before the experiment. (**D**) DLS correlation coefficient plots from two independent trials of PTDM1 at 10 μM in PBS that had been stored in solution for ~24 h prior to measurements. Each trial run was comprised of three measurements. (**E**) DLS correlation coefficient plots for three independent trials with 10 µM PTDM1 solutions in Milli-Q^®^ water. Each trial run was comprised of three measurements. Each sample was prepared immediately before the experiment. (**F**) DLS correlation coefficient plots for three independent trials with 10 µM PTDM1 solutions in Milli-Q^®^ water that had been stored in solution for ~24 h prior to measurements. Each trial run was comprised of three measurements.

Dynamic light scattering (DLS) can be used to detect the presence of self-assembled polymer aggregates and can also be used to indicate the absence of aggregates [16,33,34]. TEM and IFT provided strong indications that PTDM1 is not prone to aggregate in water at low, application-relevant concentrations, but to verify these observations, DLS and %T assays were conducted in water and also in biologically relevant salt conditions using PBS (Figure 4). %T assays have been used by our group previously to assess PTDM-protein complexation [31].

To assess the potential for PTDM1 to aggregate in PBS, transmittance experiments were conducted in which the transmittance (%T) of various wavelengths of light through PTDM1 solutions at 10 µM were monitored over the course of ~24 h (Figure 4A,B, Figures S8 and S9). UV-Visible spectra were also collected for PTDM1 at 10 µM to determine PTDM1’s absorbance profile (Figures S10 and S11). As seen in Figure 4A, for PTDM1 in PBS, the %T of 700 nm light, which PTDM1 does not inherently absorb, decreases over time compared to PTDM1 in water. This observation was suggestive of PTDM1’s ability to aggregate in PBS if given enough time. The growth in either size or number of aggregates could have accounted for the decrease in %T, but this interpretation was not definitive as a result of this technique. The decrease in %T for PTDM1 in PBS over time and relative to PTDM1 in water was also noted for smaller wavelengths of 300, 400, and 500 nm (Figure 4B, Figures S8 and S9). PTDM1 had some absorbance at the smallest wavelengths tested, but the trends over time were the same. Since %T provided an indication that aggregates could be forming, DLS measurements with PTDM1 solutions were conducted.

When DLS experiments were conducted immediately following the preparation of PTDM1 10 µM PBS solutions, the results overall indicated a lack of detectable aggregates or self-assembled structures on the basis of the poor correlation coefficient functions obtained (Figure 4C). Since the %T data seemed to indicate that aggregation could occur over time, DLS experiments were conducted again after a ~24 h waiting period with new PTDM1 solutions in PBS, during which time PTDM1 was stored in solution. For these samples, the results still indicated a lack of PTDM1 aggregates in solution (Figure 4D). The absence of aggregates was also noted for PTDM1 at 10 µM in water solutions whether DLS measurements were made immediately following sample preparation (Figure 4E) or after a ~24 h waiting period for new samples during which time PTDM1 was stored in the water solution (Figure 4F).

According to the DLS instrument manual, based on the potential size range of our polymer “particle”, which is most likely a small, single chain, and the concentration at which the experiments were conducted, there should be more than a sufficient number of “particles” or scatterers in the scattering volume of the DLS sample. Although supposedly there was a sufficient number of scatterers, they were clearly not large enough to scatter sufficient light to be detected, giving credence to the idea that PTDM1 predominantly exists as single chains in aqueous media and not aggregates. Although the %T data implies that PTDM1 could aggregate in PBS over time, this conclusion is not supported by the data obtained by DLS. The %T assay is a bulk solution measurement through the whole sample, however, which could better detect the presence of larger aggregates even if there were too few to be observed by DLS. Most importantly, the %T data seems to indicate that aggregation, if it occurs at all, only takes place over long time periods of at least 24 h. In terms of the application, protein binding and delivery experiments take place over shorter time periods: 30 min for binding/complexation and 4 h for delivery.

**Figure 5:**
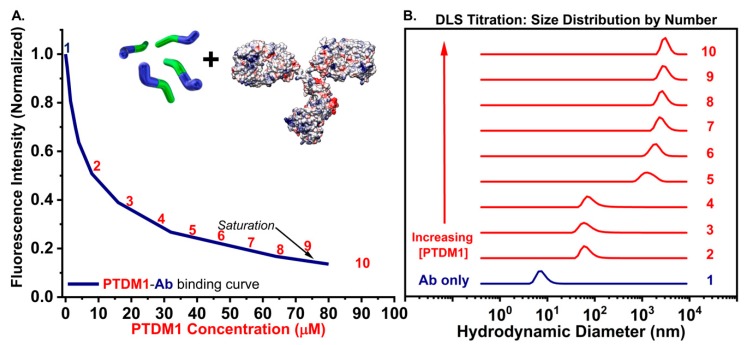
(**A**) Re-plotted Antibody (Ab)-PTDM1 fluorescence-based binding curve from reference 31 with cartoon PTDMs and a model IgG protein shown above (refer to Figure 1 for model information). (**B**) DLS-based titration of Antibody (Ab) with PTDM1 showing protein-induced complex formation at PTDM1 = ~10 µM (step 2) and growth of the complex using increasing PTDM1 concentrations like those used to produce the binding curve in part A.

Figure 5 shows the results of one trial of DLS titration experiments done with PTDM1 and IgG-FITC, where the addition of PTDM1 solution to the antibody solution resulted in a shift in the size distribution by orders of magnitude throughout the course of the titration (Figure 5B). The concentrations covered in the DLS titration can also be mapped onto similar concentration points along a previously published antibody-PTDM1 binding curve re-plotted in Figure 5A [31]. Taken together, the data show that increasing the PTDM concentration in the presence of protein results in the initiation, formation, and growth of a complex as the previously published fluorescence-quenching and %T data suggested. Figure 4 shows that PTDM1-only at 10 µM is not assembled into an aggregate detectable by DLS. However, at a similar PTDM concentration, in the presence of protein, an aggregate is formed, as evidenced by the robust correlation function and corresponding size distribution plots (Figure S12). This observation supports the idea that PTDM-protein complexes are brought about by protein-induced assembly and not self-assembly of the PTDM at delivery experiment relevant concentrations.

The DLS data of the antibody-only solutions used for both titrations are shown as a positive control to further emphasize that the correlation functions obtained for the PTDM1-only solutions were in fact noise and not indicative of a self-assembled aggregate (Figures S12 and S13). Changes in the intensity-based size distribution plots at key points in the titration are also shown in the Supplementary Materials along with all number-based size distribution plots at every step of the DLS titration for both trials, highlighting that the overall trend is reproducible (Figures S12 and S14–S18). 

## 4. Conclusions

Considering all data presented herein, PTDM1 does not aggregate or self-assemble in aqueous media at application-relevant concentrations and time scales. Therefore, PTDM1 is more likely to bind protein cargo as single chains than as aggregates. IFT and TEM together show that in simple conditions without salt, PTDM1 is a poor surfactant and cannot form aggregates aside from mere TEM grid drying artifacts. While there is some indication that PTDM1 has the potential to aggregate in PBS by %T assays, aggregation could not be confirmed by DLS. As such, PTDM1 cannot readily form robust aggregates capable of being detected by DLS furthering the conclusion that PTDM1 is not inclined to aggregate or self-assemble prior to binding protein cargo in PBS. We argue instead that PTDMs more likely bind protein cargo as single chains. Therefore, in line with the findings of our related studies and with the results of the DLS experiments conducted within, the presence of protein in solution with the PTDM is required to induced complex formation. Future studies could elucidate whether the lack of self-assembly behavior applies to additional PTDMs with other side chains or higher molecular weights. Additionally, protein-induced complex assembly could be studied for other combinations of PTDMs and protein cargoes to assess the general applicability of this observation.

## Supplementary Materials

The following are available online at http://www.mdpi.com/2073-4360/10/9/1039/s1, Figure S1: Three independent trials of pendant drop interfacial tension (IFT) raw data for Milli-Q^®^ water droplet in a Toluene ambient phase, Figure S2: Three independent trials of pendant drop interfacial tension (IFT) raw data for Milli-Q^®^ water with 10% *v*/*v* DMSO droplet in a Toluene ambient phase, Figure S3: Three independent trials of pendant drop interfacial tension (IFT) raw data for PTDM1 1 µM in Milli-Q^®^ water with 10% *v*/*v* DMSO droplet in a Toluene ambient phase, Figure S4: Two independent trials of pendant drop interfacial tension (IFT) raw data for PTDM1 10 µM in Milli-Q^®^ water with 10% *v*/*v* DMSO droplet in a Toluene ambient phase, Figure S5: Two independent trials of pendant drop interfacial tension (IFT) raw data for PTDM1 100 µM in Milli-Q^®^ water with 10% *v*/*v* DMSO droplet in a Toluene ambient phase, Figure S6: Comparison of surfactants from literature (SDS and PS-PEO) and PTDM1 with respect to their ability to decrease IFT, denoted as ΔIFT, at a water-toluene interface, Figure S7: TEM micrograph of 1× PBS solution with 1% *v*/*v* DMSO that was solvent cast onto a TEM grid followed by removal of excess liquid and drying at ambient conditions overnight, Figure S8: %T of 300 nm light through solutions containing PTDM1 at 10 µM in both Milli-Q^®^ water (blue squares) and PBS (red circles), Figure S9: %T of 500 nm light through solutions containing PTDM1 at 10 µM in both Milli-Q^®^ water (blue squares) and PBS (red circles), Figure S10: UV-visible spectra of PTDM1 in water and PBS along with control solutions of water-only and PBS-only, Figure S11: UV-visible spectra of PTDM1 in water and PBS along with control solutions of water-only and PBS-only, Figure S12: Dynamic Light Scattering (DLS) titration experiment representative overview showing: (A) Correlation coefficient function for PTDM1-only at 10 µM in PBS (shown again for comparison), Figure S13: Dynamic Light Scattering (DLS) data showing correlation coefficient functions for IgG-FITC antibody (Ab) at 200 nM in PBS with their corresponding intensity-based size distributions, Figure S14: DLS titration data from a second trial of the DLS titration experiment showing correlation functions (left), individual intensity-based size distributions from the results of the three measurements taken per sample (middle), and the averaged intensity-based size distributions shown with ± one standard deviation obtained from the three individual measurements taken of the sample per this second trial, Figure S15: DLS titration data showing number-based size distribution plots (3 measurements per sample) for the starting solution of antibody-only for the two trials, Figure S16: DLS titration data showing number-based size distribution plots (3 measurements per sample) for each step of the titration where the total volume of PTDM1 1 mM DMSO stock solution that had been added up to that point is shown above the plot for both trials, Figure S17: DLS titration data showing number-based size distribution plots (3 measurements per sample) for each step of the titration where the total volume of PTDM1 1 mM DMSO stock solution that had been added up to that point is shown above the plot for both trials, Figure S18: DLS titration data showing number-based size distribution plots (3 measurements per sample) for each step of the titration where the total volume of PTDM1 1 mM DMSO stock solution that had been added up to that point is shown above the plot for both trials., Table S1: IFT values for PTDM1 and surfactants from literature used for creating comparison plots, Table S2: DLS titration experiment concentrations and additions summary table.
