# Supplementary material for: Protein Transduction Domain Mimic (PTDM) Self-Assembly?
Source: Polymers (Basel). 2018 Sep 19;10(9):1039. doi: 10.3390/polym10091039 (PMC6403535; doi:10.3390/polym10091039)
Supplement: Supplementary file 1 [file polymers-10-01039-s001.pdf]

SUPPLEMENTARY MATERIALS

Protein Transduction Domain Mimic (PTDM) Self-assembly?

Nicholas D. Posey[†], Prof. Gregory N. Tew[*†,§,‡]

[†]Department of Polymer Science and Engineering, University of Massachusetts Amherst, Amherst, MA 01003
[§]Department of Veterinary and Animal Sciences, University of Massachusetts Amherst, Amherst, MA 01003
[‡]Molecular and Cellular Biology Program, University of Massachusetts Amherst, Amherst, MA 01003

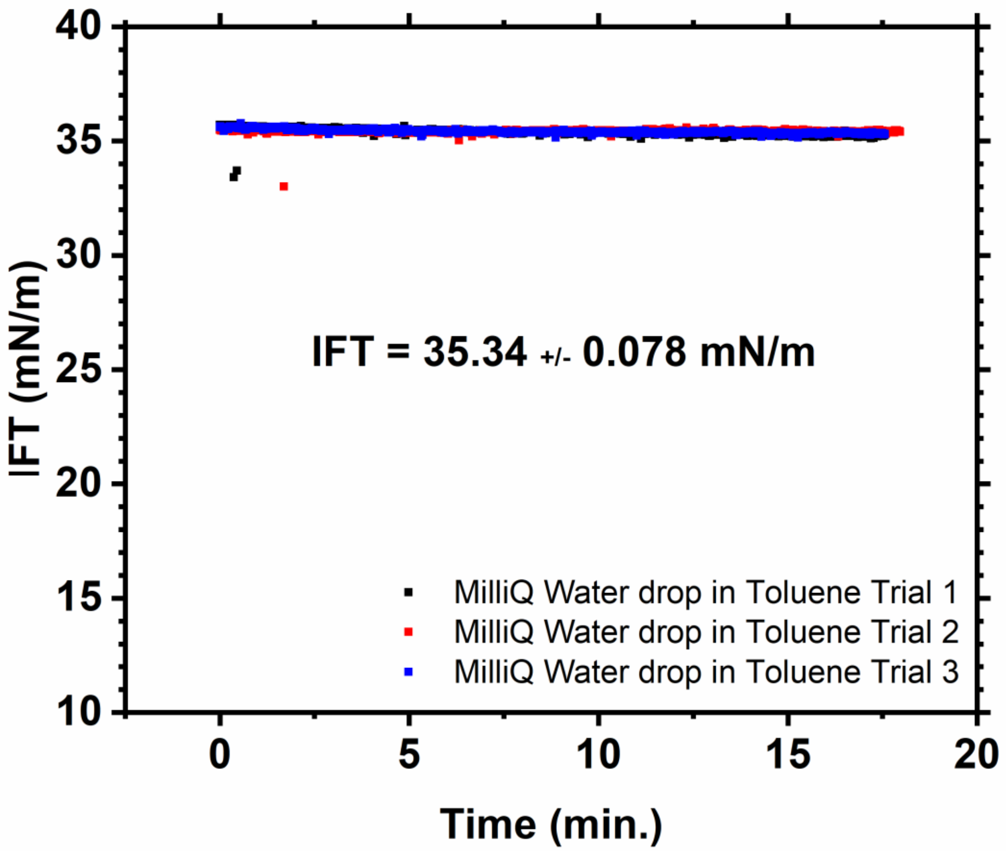

Figure S1. Three independent trials of pendant drop interfacial tension (IFT) raw data for Milli-Q® water droplet in a Toluene ambient phase. All data points from minutes 12 through 17 from all three trials were averaged together to obtain one average value with +/- one standard deviation shown as an inset.

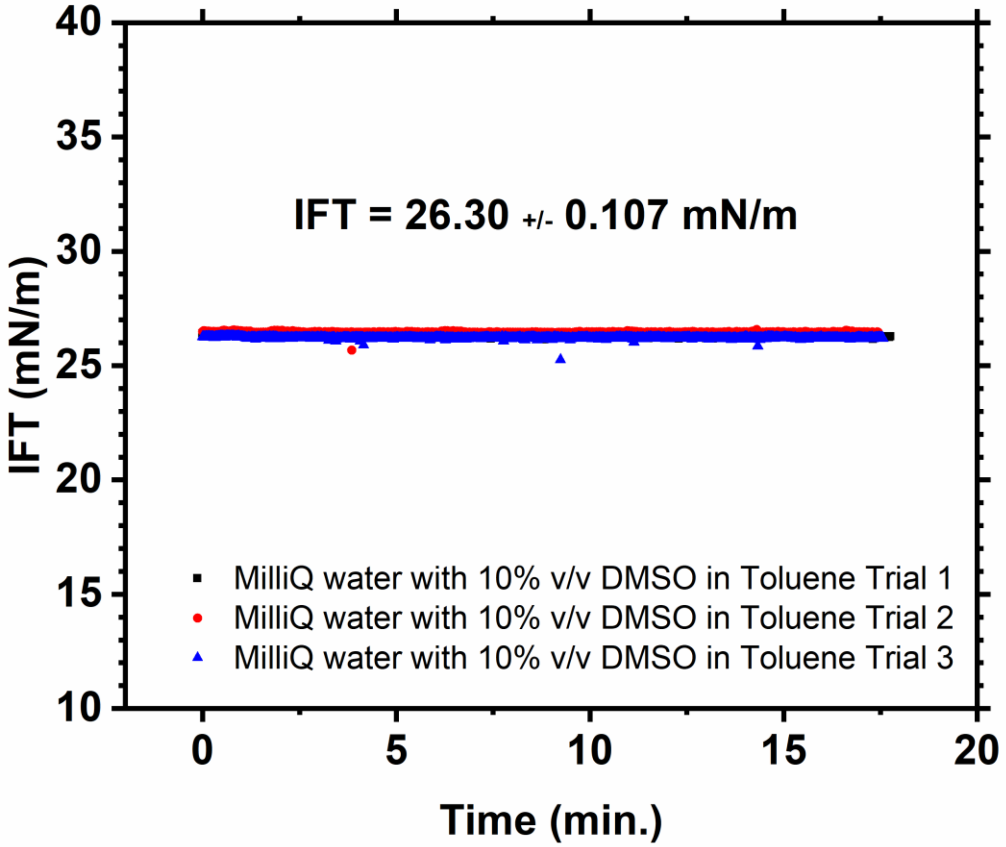

Figure S2. Three independent trials of pendant drop interfacial tension (IFT) raw data for Milli-Q® water with 10% v/v DMSO droplet in a Toluene ambient phase. All data points from minutes 12 through 17 from all three trials were averaged together to obtain one average value with +/- one standard deviation shown as an inset.

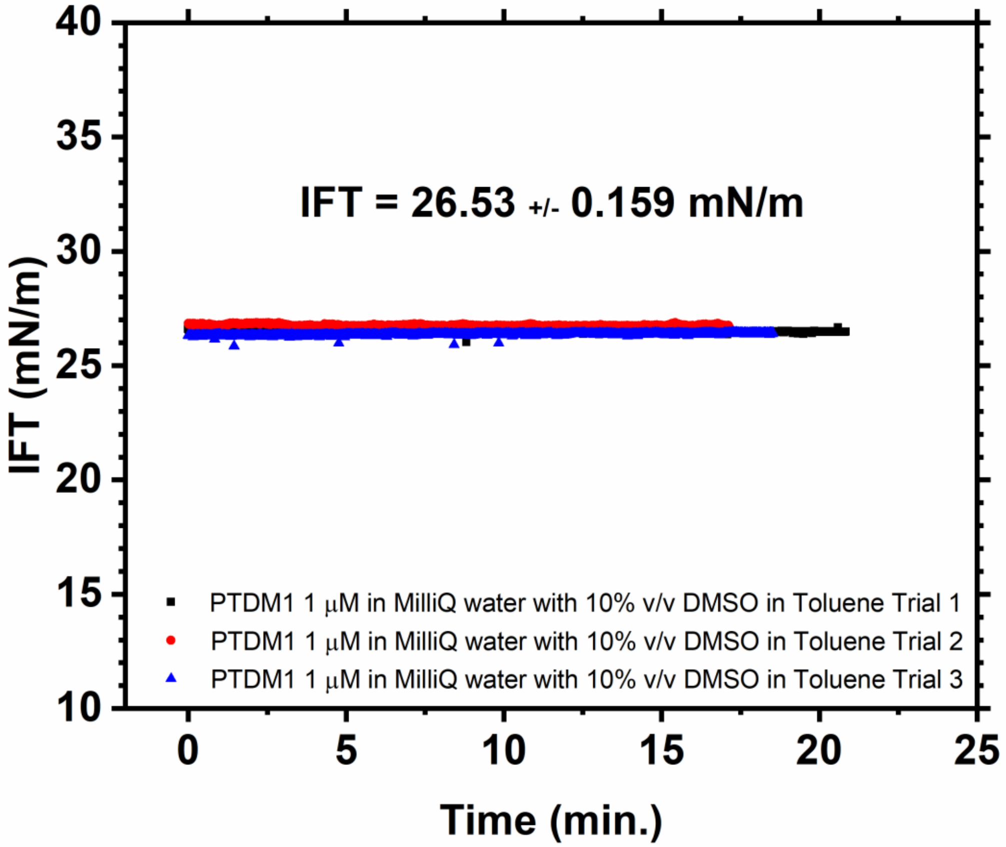

Figure S3. Three independent trials of pendant drop interfacial tension (IFT) raw data for PTDM1 1 µM in Milli-Q® water with 10% v/v DMSO droplet in a Toluene ambient phase. All data points from minutes 12 through 17 from all three trials were averaged together to obtain one average value with +/- one standard deviation shown as an inset.

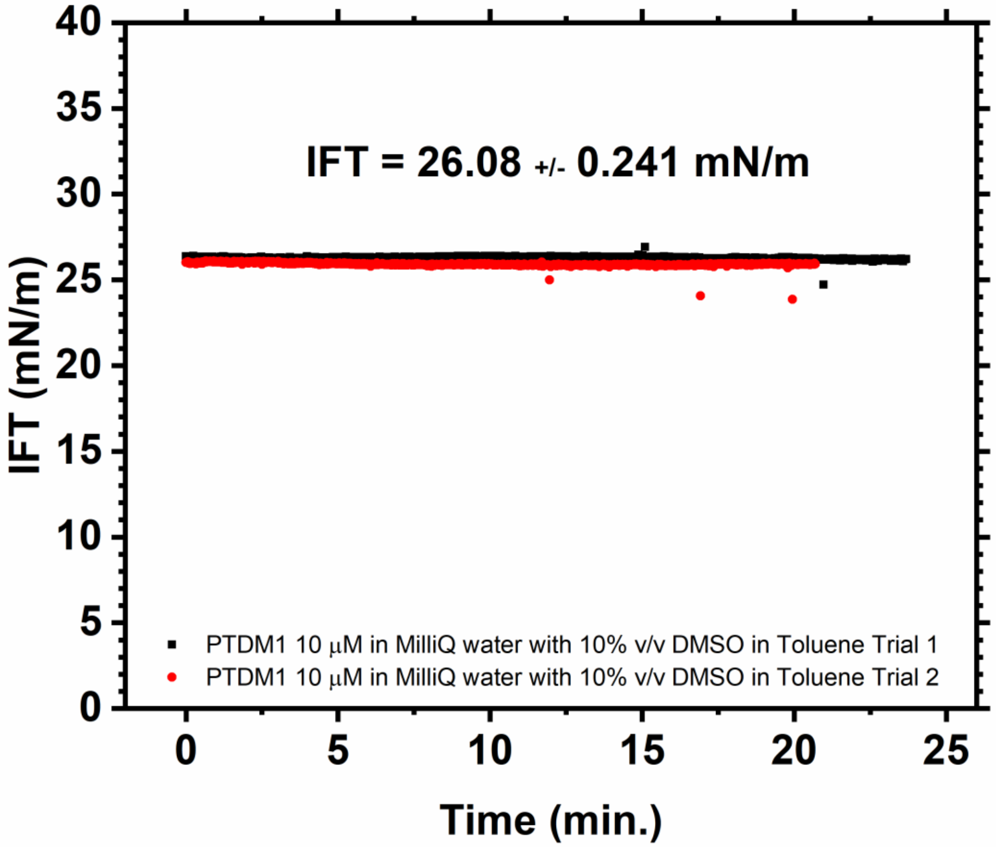

Figure S4. Two independent trials of pendant drop interfacial tension (IFT) raw data for PTDM1 10 μM in Milli-Q® water with 10% v/v DMSO droplet in a Toluene ambient phase. All data points from minutes 12 through 17 from both trials were averaged together to obtain one average value with +/- one standard deviation shown as an inset.

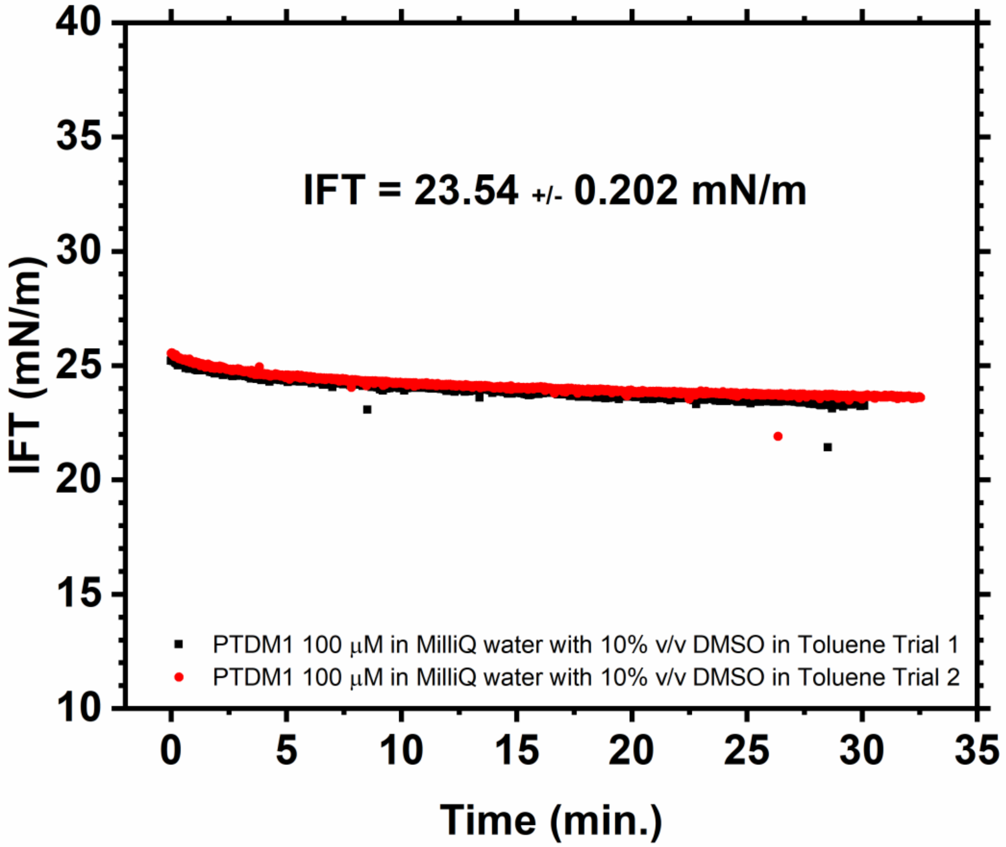

Figure S5. Two independent trials of pendant drop interfacial tension (IFT) raw data for PTDM1 100 µM in Milli-Q® water with 10% v/v DMSO droplet in a Toluene ambient phase. All data points from minutes 25 through 30 from both trials were averaged together to obtain one average value with +/- one standard deviation shown as an inset.

Table S1. IFT values for PTDM1 and surfactants from literature used for creating comparison plots.

**Surfactant (conditions)**	**Sample Concentration**	**IFT (mN/m)[a]**	**ΔIFT (mN/m)[b]**
PTDM1 (pH 4-5, RT)	0 µM (control with 10% DMSO v/v)	26.30	0
	1 µM	26.53	0.230
	10 µM	26.08	-0.230
	100 µM	23.54	-2.77
Sodium Dodecyl Sulfate (pH 4, 20 °C)[1]	0 µM (control)	38.1	0
	21.7 µM	36.5	-1.6
	43.3 µM	35	-3.1
	86.7 µM	33.1	-5
Sodium Dodecyl Sulfate (pH 4, 25 °C)[1]	0 µM (control)	37.5	0
	21.7 µM	35.7	-1.8
	43.3 µM	34.4	-3.1
	86.7 µM	32.7	-4.8
Sodium Dodecyl Sulfate (pH 5, 20 °C)[1]	0 µM (control)	37.8	0
	21.7 µM	36.2	-1.6
	43.3 µM	34.8	-3
	86.7 µM	32.8	-5
Sodium Dodecyl Sulfate (pH 5, 25 °C)[1]	0 µM (control)	37.1	0
	21.7 µM	35.5	-1.6
	43.3 µM	34.2	-2.9
	86.7 µM	32.2	-4.9
PS-PEO (252,070 g/mol, 20 °C)[2]	0 µM (control)	35.3	0
	0.520 µM	27.8	-7.5
Bu-PPO-PEO (900 g/mol, 25 °C)[3]	0 µM (control)	32	0
	11.1 mM	8.1	-23.9
Bu-PEO-PPO (920 g/mol, 25 °C)[3]	0 µM (control)	32	0
	10.9 mM	11.5	-20.5
PEO-PPO-PEO (1,460 g/mol, 25 °C)[3]	0 µM (control)	32	0
	6.85 mM	12.1	-19.9

[a]Interfacial Tension of a water-toluene interface. [b]ΔIFT was calculated as $IFT_{Sample} - IFT_{Control}$ for all surfactants at all concentrations.

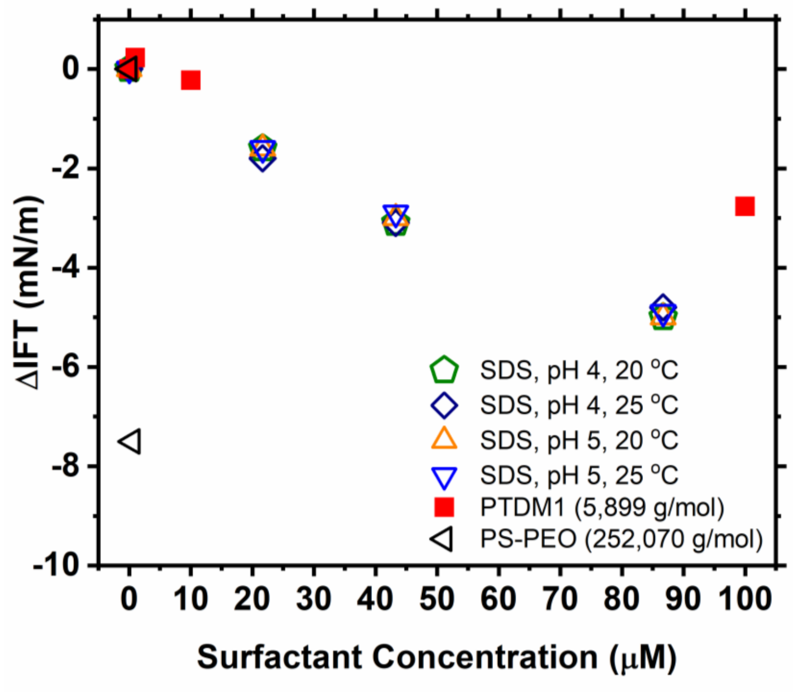

Figure S6. Comparison of surfactants from literature (SDS and PS-PEO) and PTDM1 with respect to their ability to decrease IFT, denoted as ΔIFT, at a water-toluene interface. SDS is shown with respect to various pH's and temperatures in this version of the plot.

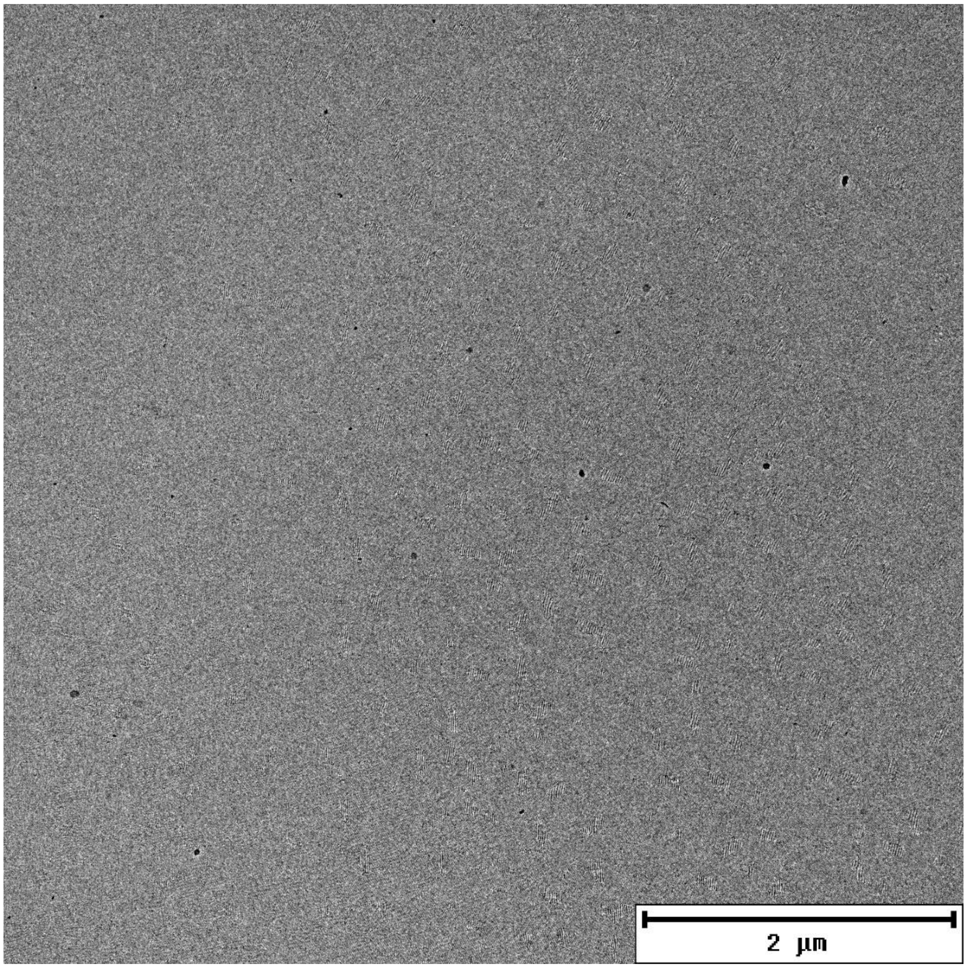

Figures S7. TEM micrograph of 1X PBS solution with 1% v/v DMSO that was solvent cast onto a TEM grid followed by removal of excess liquid and drying at ambient conditions overnight. Image was taken at 1.25 µm under focus.

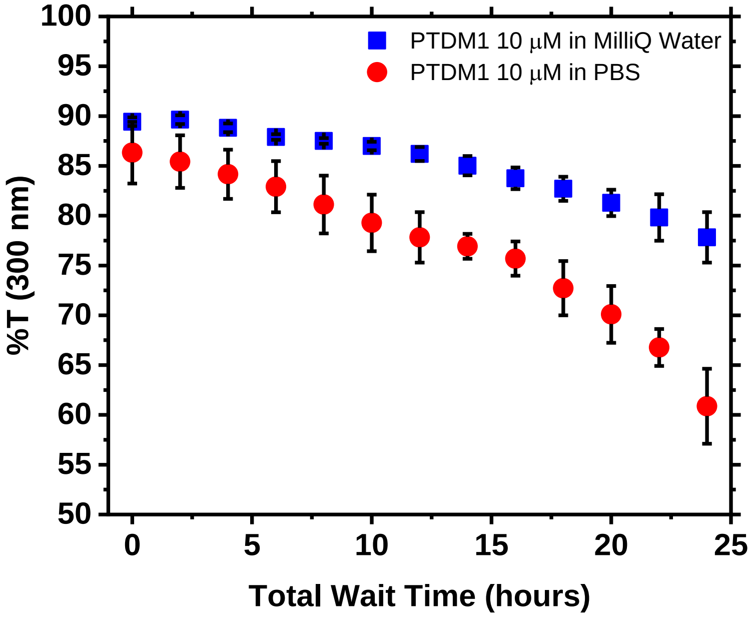

Figure S8. %T of 300 nm light through solutions containing PTDM1 at 10 µM in both Milli-Q® water (blue squares) and PBS (red circles). Results obtained are the average of two independent trials where the average %T is plotted and the error bars represent ± one standard deviation.

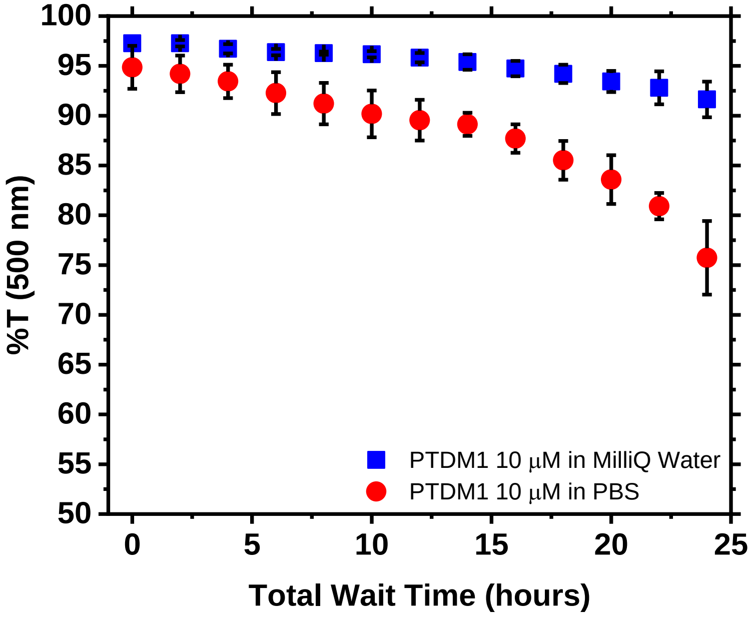

Figure S9. %T of 500 nm light through solutions containing PTDM1 at 10 µM in both Milli-Q® water (blue squares) and PBS (red circles). Results obtained are the average of two independent trials where the average %T is plotted and the error bars represent ± one standard deviation.

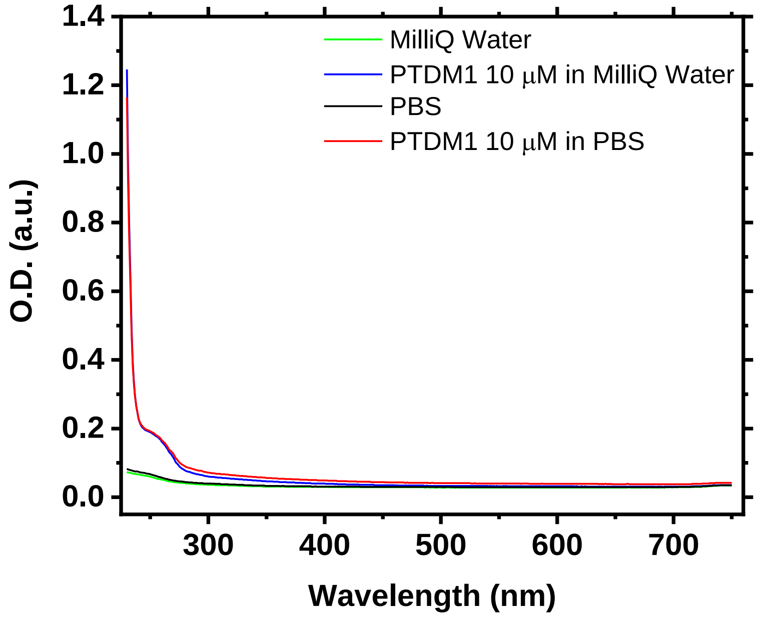

Figure S10. UV-visible spectra of PTDM1 in water and PBS along with control solutions of water-only and PBS-only. Results from one trial independent trial.

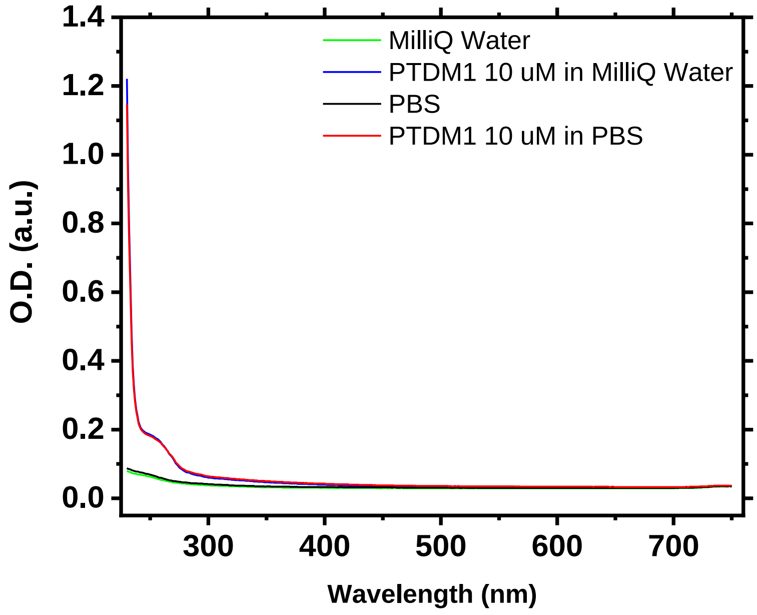

Figure S11. UV-visible spectra of PTDM1 in water and PBS along with control solutions of water-only and PBS-only. Results from a second independent trial.

DLS Titration Experiments

Both of the following titration experiments utilized Malvern's "protein" option as the "material" instrument setting, even though PTDM1 was being added. This was because the "protein" preset option (RI (refractive index) = 1.45 and absorbance = 0.001) came from Malvern whereas the "polynorbornene" preset was made by another user and could not be verified on the Malvern website. Regardless, the RI and absorbance parameters were used in Mie theory to convert the intensity-based size distributions to number-based size distributions. Size numbers are not reported as they are only approximate being based on light scattering from only one angle and given that the solution viscosity, RI, and absorbance parameters are approximate. In order to do this titration experiment, several assumptions were made: 1) the calculations of PTDM concentration were based on the assumption that there was still close to 1 full milliliter of the antibody-only solution after the one and only initial filtering step. 2) DMSO concentrations increased throughout the titration up to 9% DMSO v/v by the final addition, but those concentrations did not drive complex formation or cause the antibody to precipitate because in the corresponding %T assay, an antibody-only well with 200 nM antibody and 8% DMSO was still close to 100% transparent. 3) The pure PTDM solution and the starting antibody solution was only filtered once in the beginning of the titration and any dust that may have been introduced by the many additions of PTDM would be caught by the dust filter algorithm of the instrument if in fact dust was present and scattering large amounts of light.

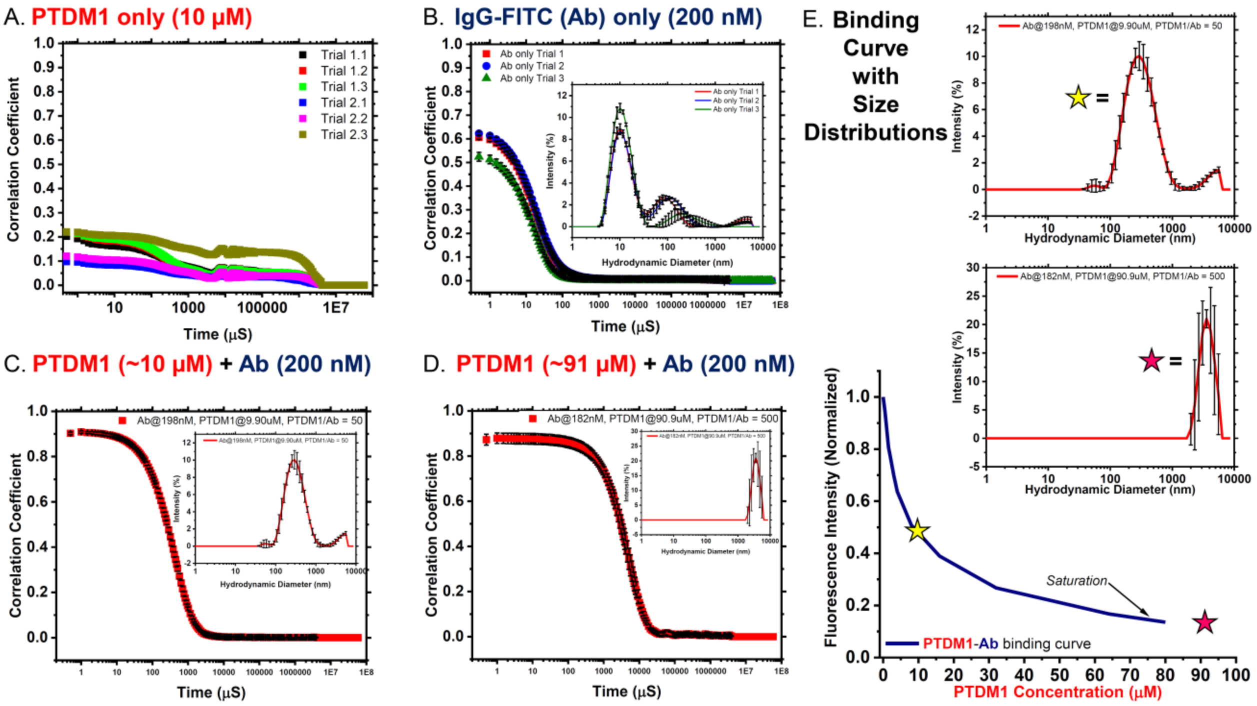

Figure S12. Dynamic Light Scattering (DLS) titration experiment representative overview showing: A) Correlation coefficient function for PTDM1-only at 10 μM in PBS (shown again for comparison); B) Three independent trials of correlation coefficient functions for IgG-FITC antibody (Ab) at 200 nM in PBS with the intensity-based size distributions as an inset; C) Correlation coefficient function for PTDM1 at ~10 μM + Ab at 200 nM in PBS with inset showing the intensity-based size distribution revealing the presence of an aggregate; D) Correlation coefficient function for PTDM1 at ~91 μM + Ab at 200 nM in PBS with inset showing the intensity-based size distribution revealing the presence of an larger aggregate. E) Re-plotted PTDM1-Ab binding curve from previously published data [4] with results of DLS titration experiment mapped onto concentration points along the binding curve, showing that an aggregate initiates and grows as the original fluorescence data suggests. All error bars represent ± one standard deviation obtained from the three measurements taken per sample. For parts C, D, and E data comes from first trial of DLS titration experiment.

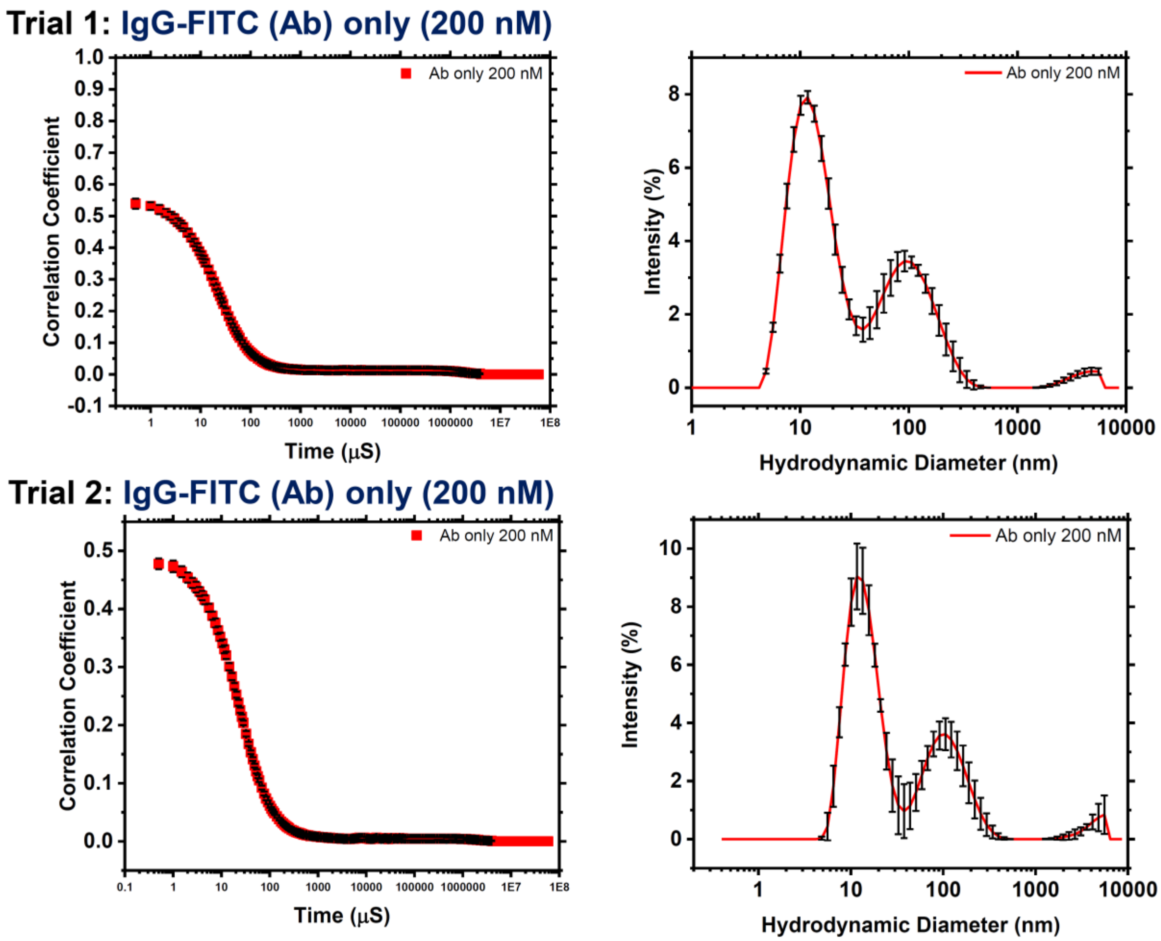

Figure S13. Dynamic Light Scattering (DLS) data showing correlation coefficient functions for IgG-FITC antibody (Ab) at 200 nM in PBS with their corresponding intensity-based size distributions. These plots represented the starting points for the two independent trials of the DLS PTDM1-Ab titration. Error bars represent ± one standard deviation obtained from the three measurements taken of the sample per trial.

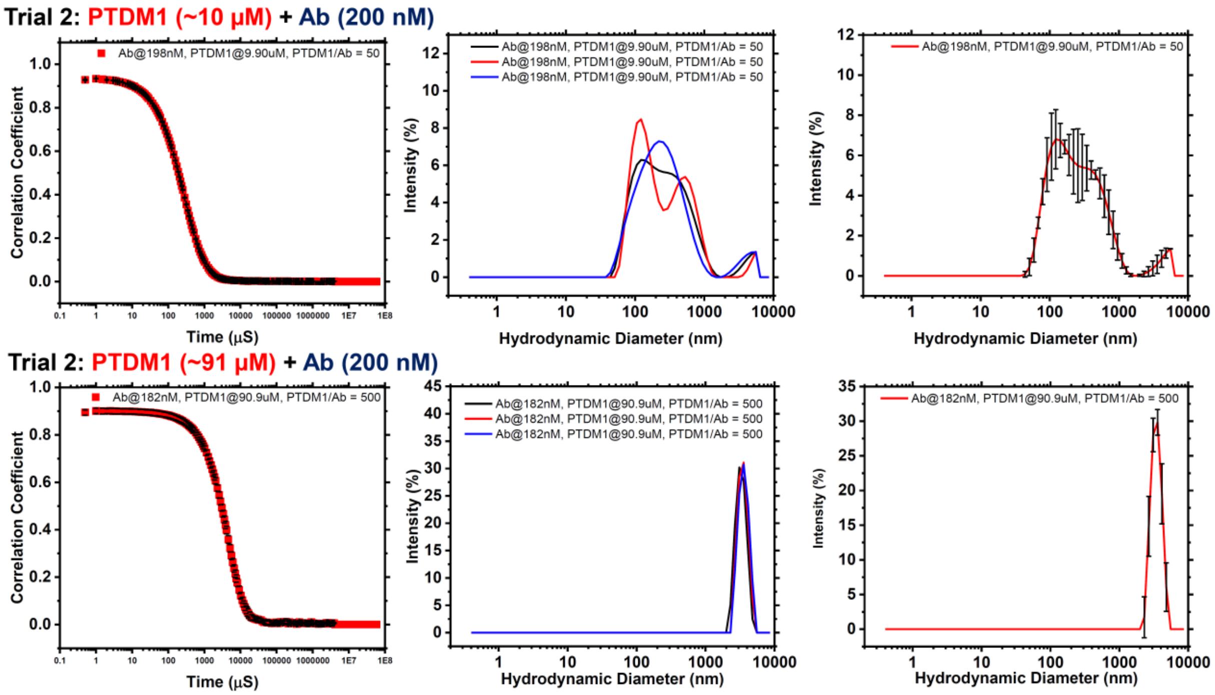

Figure S14. DLS titration data from a second trial of the DLS titration experiment showing correlation functions (left), individual intensity-based size distributions from the results of the three measurements taken per sample (middle), and the averaged intensity-based size distributions shown with ± one standard deviation obtained from the three individual measurements taken of the sample per this second trial.

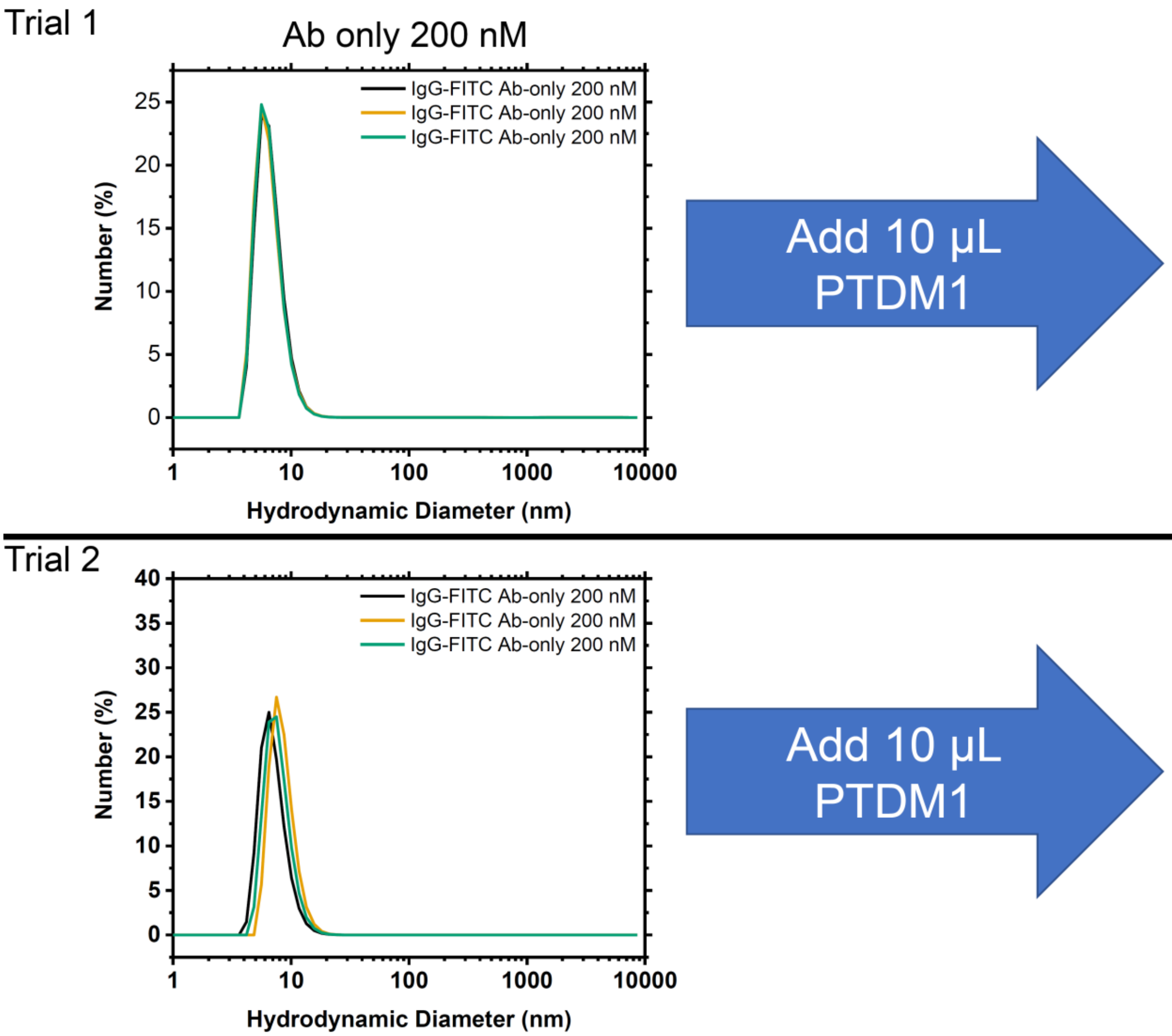

Figure S15. DLS titration data showing number-based size distribution plots (3 measurements per sample) for the starting solution of antibody-only for the two trials.

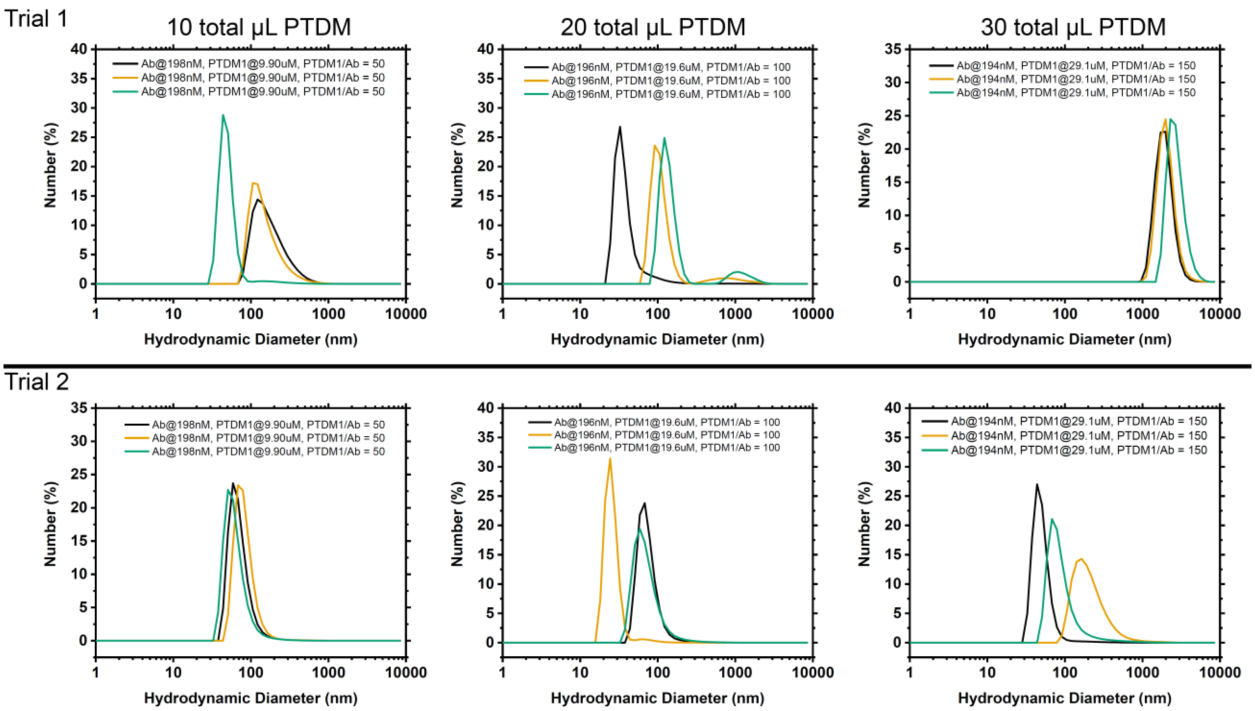

Figure S16. DLS titration data showing number-based size distribution plots (3 measurements per sample) for each step of the titration where the total volume of PTDM1 1 mM DMSO stock solution that had been added up to that point is shown above the plot for both trials.

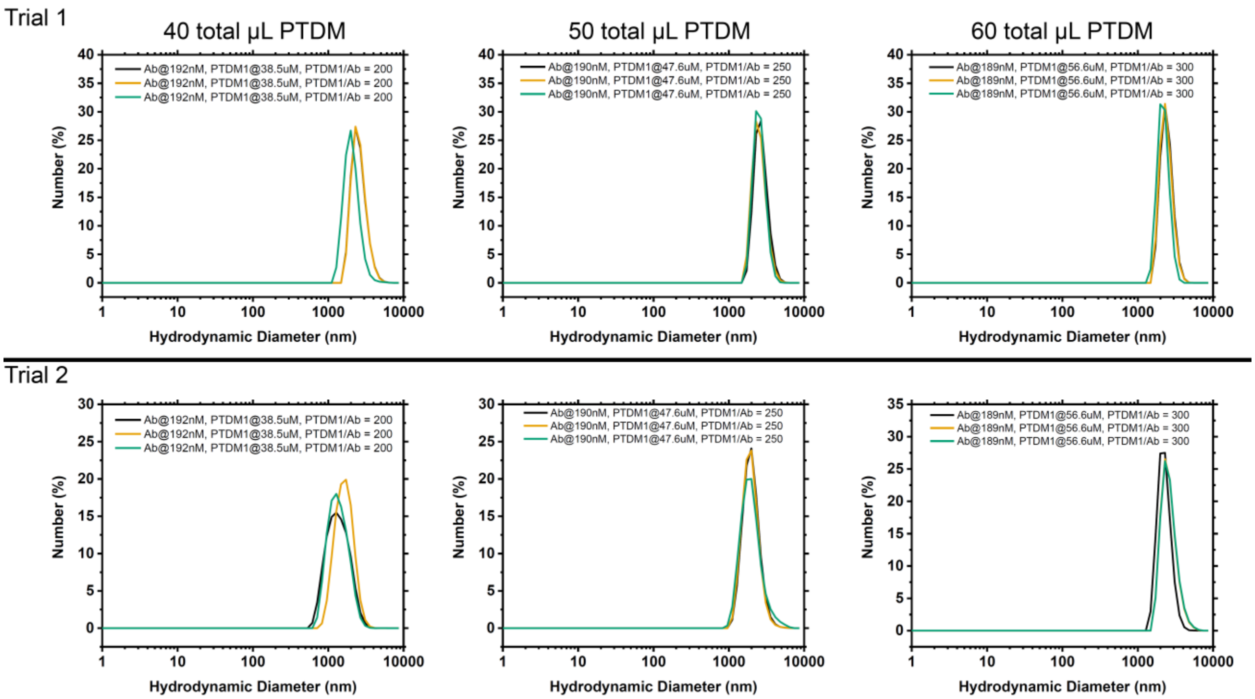

Figure S17. DLS titration data showing number-based size distribution plots (3 measurements per sample) for each step of the titration where the total volume of PTDM1 1 mM DMSO stock solution that had been added up to that point is shown above the plot for both trials.

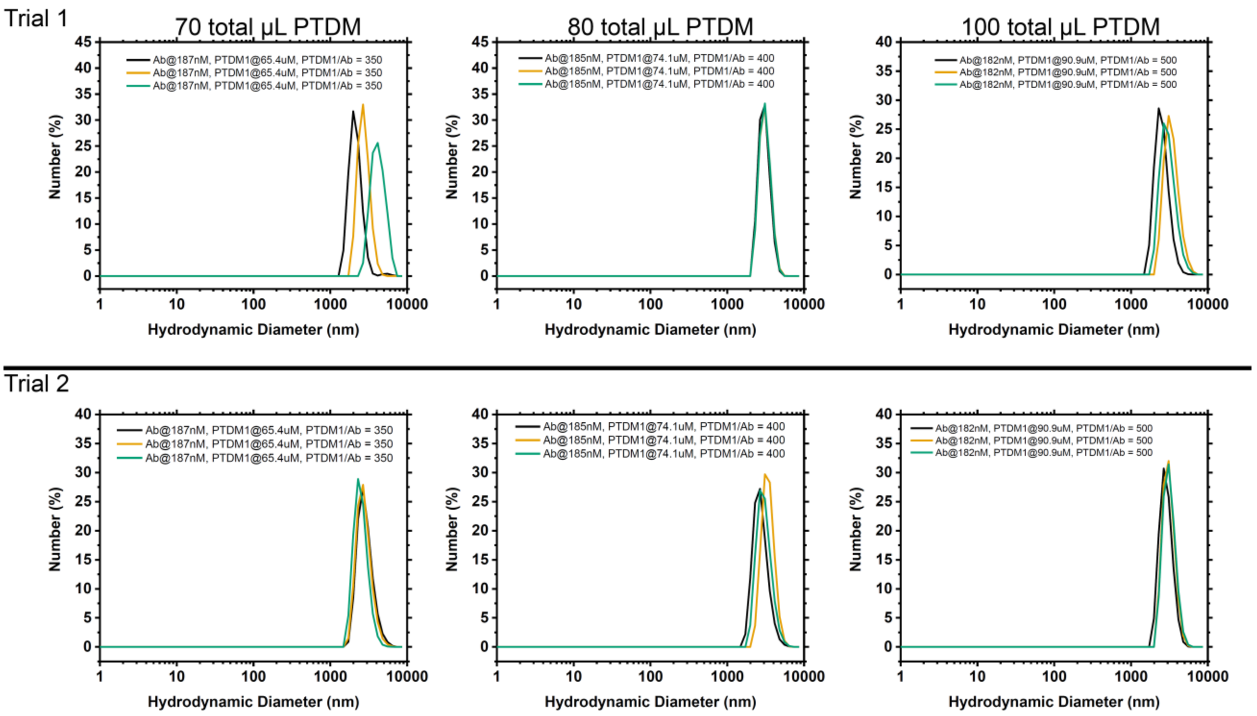

Figure S18. DLS titration data showing number-based size distribution plots (3 measurements per sample) for each step of the titration where the total volume of PTDM1 1 mM DMSO stock solution that had been added up to that point is shown above the plot for both trials.

Table S2. DLS titration experiment concentrations and additions summary table.

Total added PTDM1 (µL)	[Antibody] (nM)	[PTDM1] (µM)	Total approximate volume in cuvette (µL)	[PTDM]/[Ab] molar ratio	%DMSO (v/v)
0	200	0	1000	0	0
10	198	9.90	1010	50	0.99
20	196	19.6	1020	100	1.96
30	194	29.1	1030	150	2.91
40	192	38.5	1040	200	3.85
50	190	47.6	1050	250	4.76
60	189	56.6	1060	300	5.66
70	187	65.4	1070	350	6.54
80	185	74.1	1080	400	7.41
100	182	90.9	1100	500	9.09

NOTE: “total added PTDM1” refers to the total amount of PTDM1 solution that had been added up to that particular point in the titration. PTDM1 was added 10 µL at a time except for the final added aliquot of 20 µL.

REFERENCES

1. Saien, J.; Akbari, S. Interfacial Tension of Toluene + Water + Sodium Dodecyl Sulfate from (20 to 50) °C and pH between 4 and 9. *J. Chem. Eng. Data* **2006**, *51*, 1832–1835, doi:10.1021/je060204g.

2. Prokop, R. M.; Hair, M. L.; Neumann, A. W. Interfacial Tension of a Polystyrene-Poly(ethylene oxide) Diblock Copolymer at the Water-Toluene Interface. *Macromolecules* **1996**, *29*, 5902–5906, doi:10.1021/ma960230r.

3. Lucas, E. F.; Oliveira, C. M.; Gomes, A. S. Surface Properties of Graft Copolymers Surfactants: Behavior at the Water/Toluene Interface. *J. Appl. Polym. Sci.* **1992**, *46*, 733–737, doi:10.1002/app.1992.070460420.

4. Posey, N. D.; Hango, C. R.; Minter, L. M.; Tew, G. N. The Role of Cargo Binding Strength in Polymer-Mediated Intracellular Protein Delivery. *Bioconjugate Chem.* **2018**, *29*, 2679–2690, doi:10.1021/acs.bioconjchem.8b00363.
